# Supplementary material for: Isopsoralen suppresses receptor activator of nuclear factor kappa-β ligand-induced osteoclastogenesis by inhibiting the NF-κB signaling
Source: PeerJ. 2023 Jan 10;11:e14560. doi: 10.7717/peerj.14560 (PMC9838210; doi:10.7717/peerj.14560)

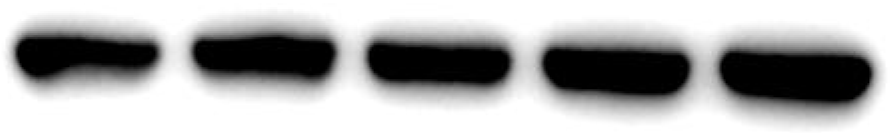

Supplement: Supplemental Information 2 [file peerj-11-14560-s002.zip › All raw data(including WB data) version 2/FIGURE 2/C(All raw WB data)/4 GAPDH.pdf]
