# Supplementary figures and images for: Isopsoralen suppresses receptor activator of nuclear factor kappa-β ligand-induced osteoclastogenesis by inhibiting the NF-κB signaling
Source: PeerJ. 2023 Jan 10;11:e14560. doi: 10.7717/peerj.14560 (PMC9838210; doi:10.7717/peerj.14560)

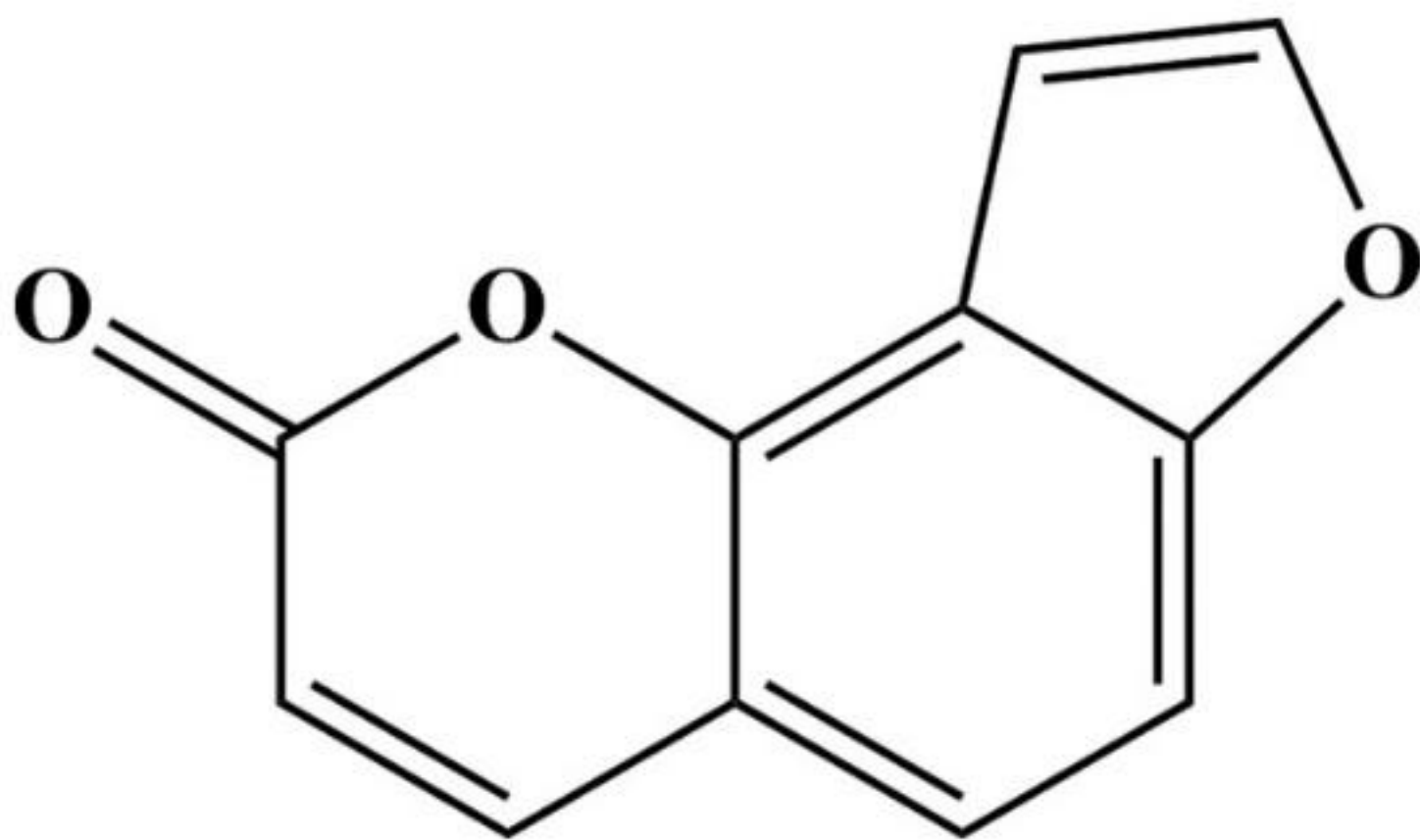

Supplement: Supplemental Information 2 [file peerj-11-14560-s002.zip › All raw data(including WB data) version 2/FIGURE 1/A/FIGURE 1 A.pdf]

24H

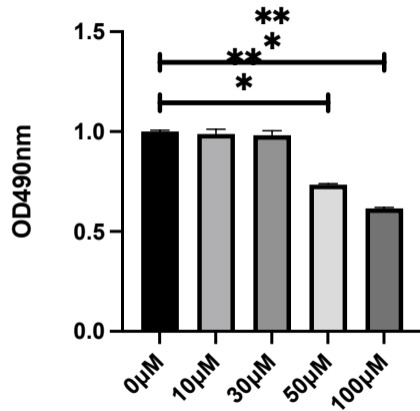

48H

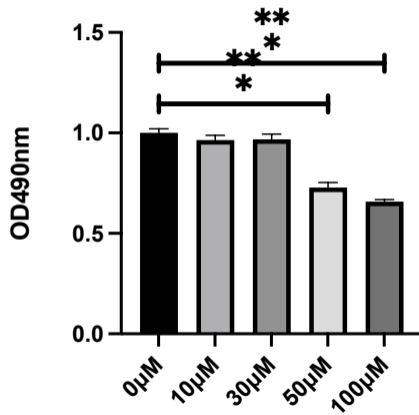

72H

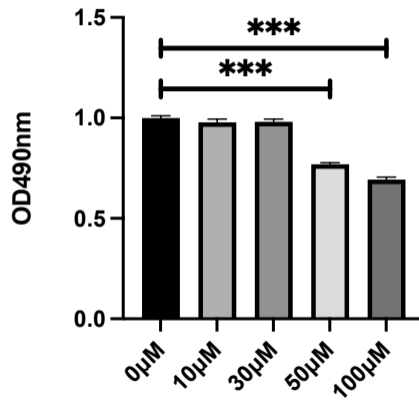

Supplement: Supplemental Information 2 [file peerj-11-14560-s002.zip › All raw data(including WB data) version 2/FIGURE 1/B/FIGURE 1 B.pdf]

**RANKL(50ng/ml)**

**RANKL(-)**

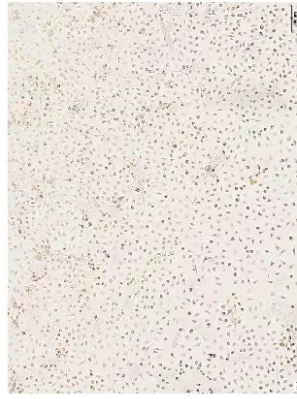

**0 $\mu$ M**

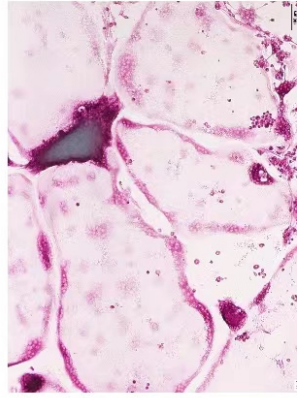

**10 $\mu$ M**

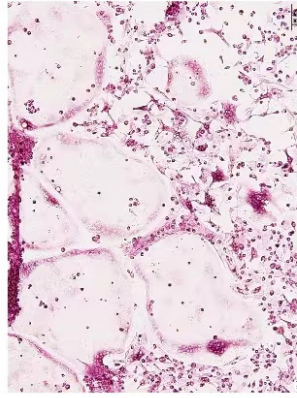

**20 $\mu$ M**

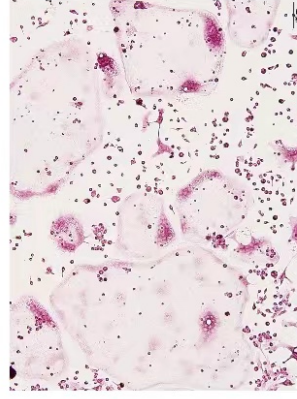

**30 $\mu$ M**

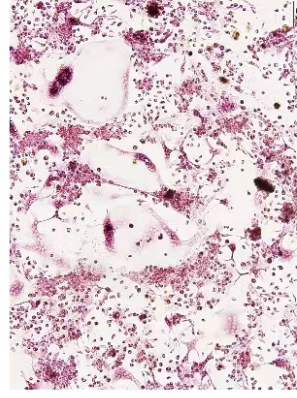

Supplement: Supplemental Information 2 [file peerj-11-14560-s002.zip › All raw data(including WB data) version 2/FIGURE 2/A/FIGURE 2 A.pdf]

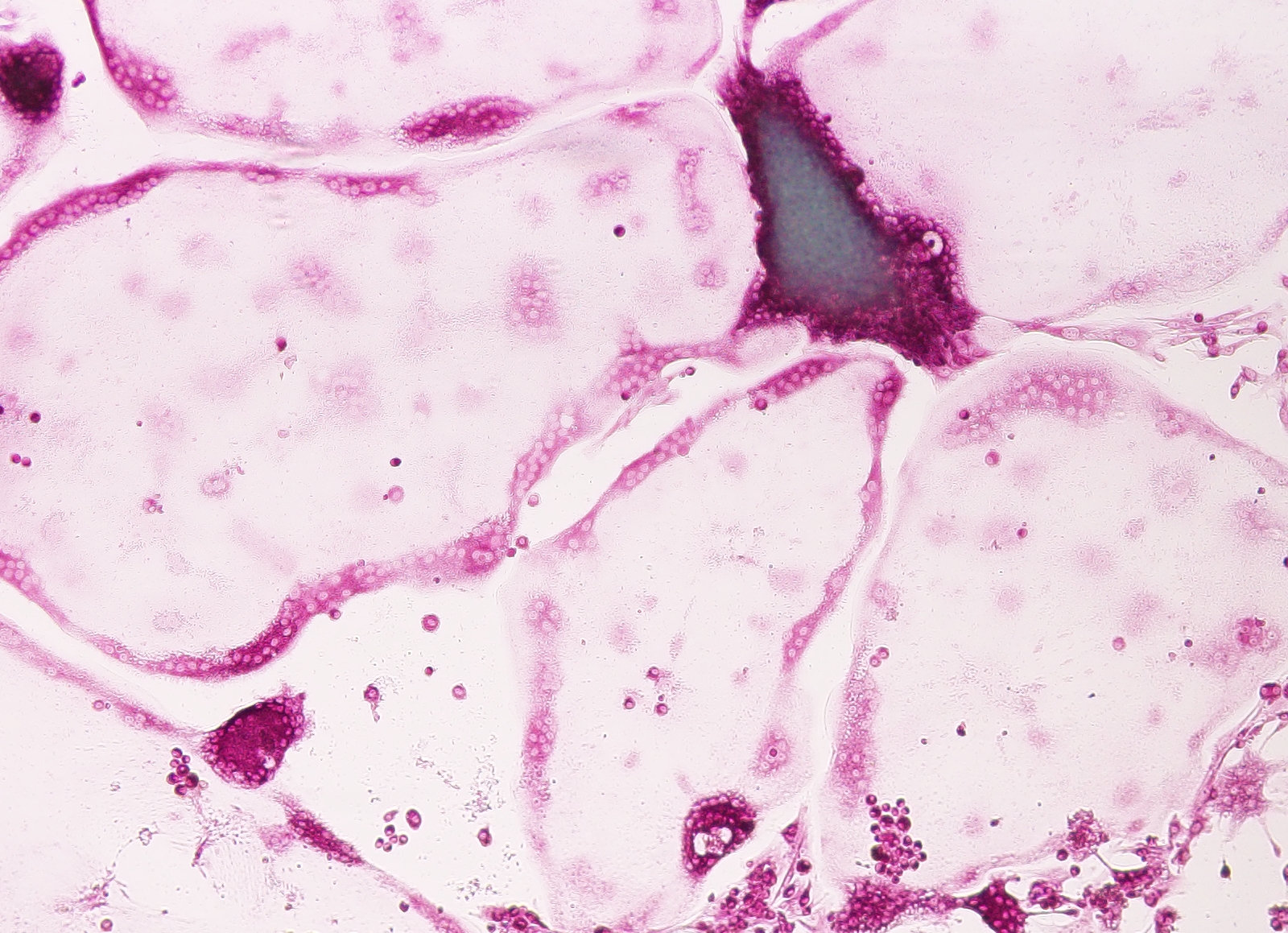

Supplement: Supplemental Information 2 [file peerj-11-14560-s002.zip › All raw data(including WB data) version 2/FIGURE 2/A/RANKL(+)-I(0).jpg]

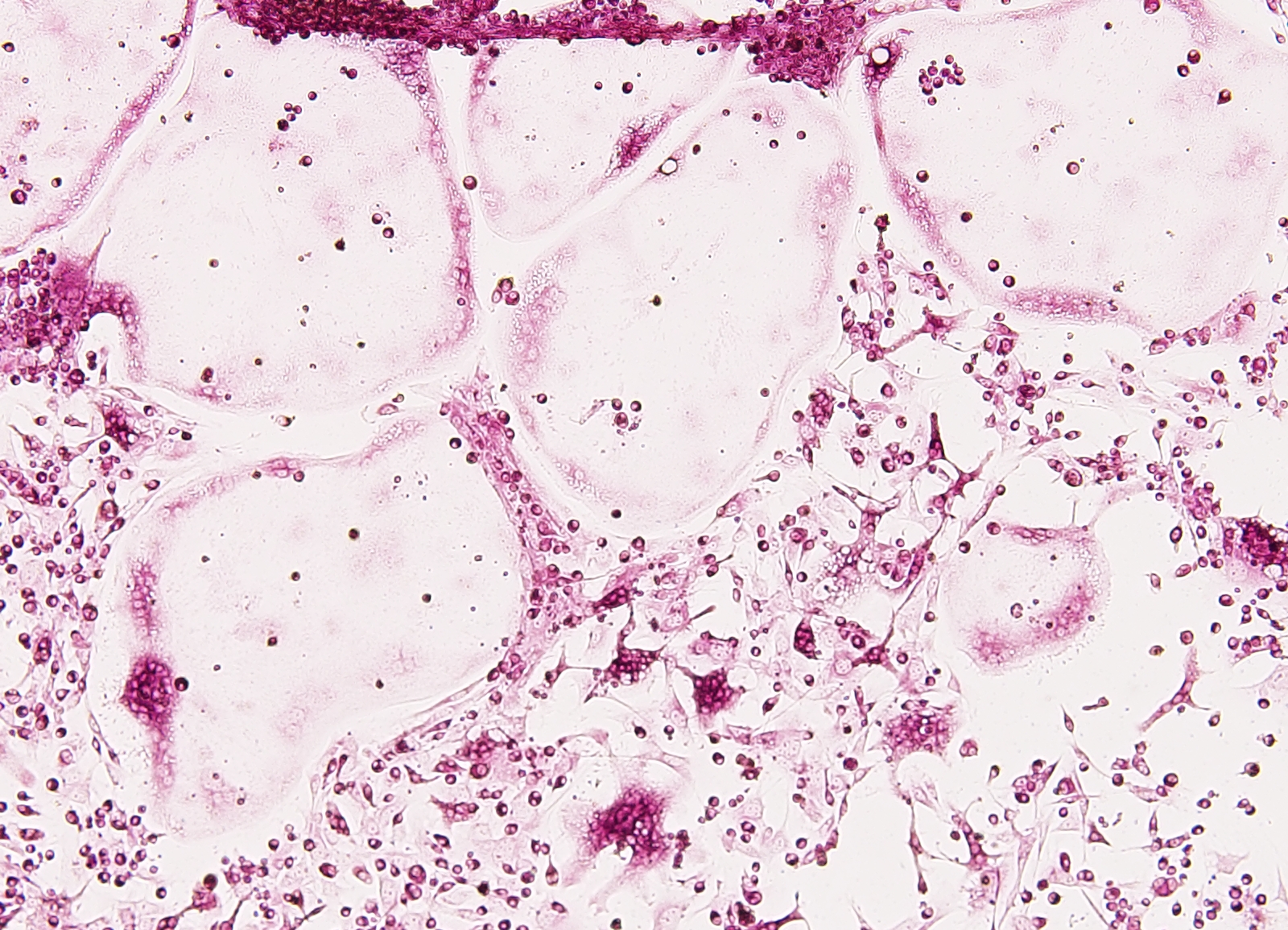

Supplement: Supplemental Information 2 [file peerj-11-14560-s002.zip › All raw data(including WB data) version 2/FIGURE 2/A/RANKL(+)-I(10).jpg]

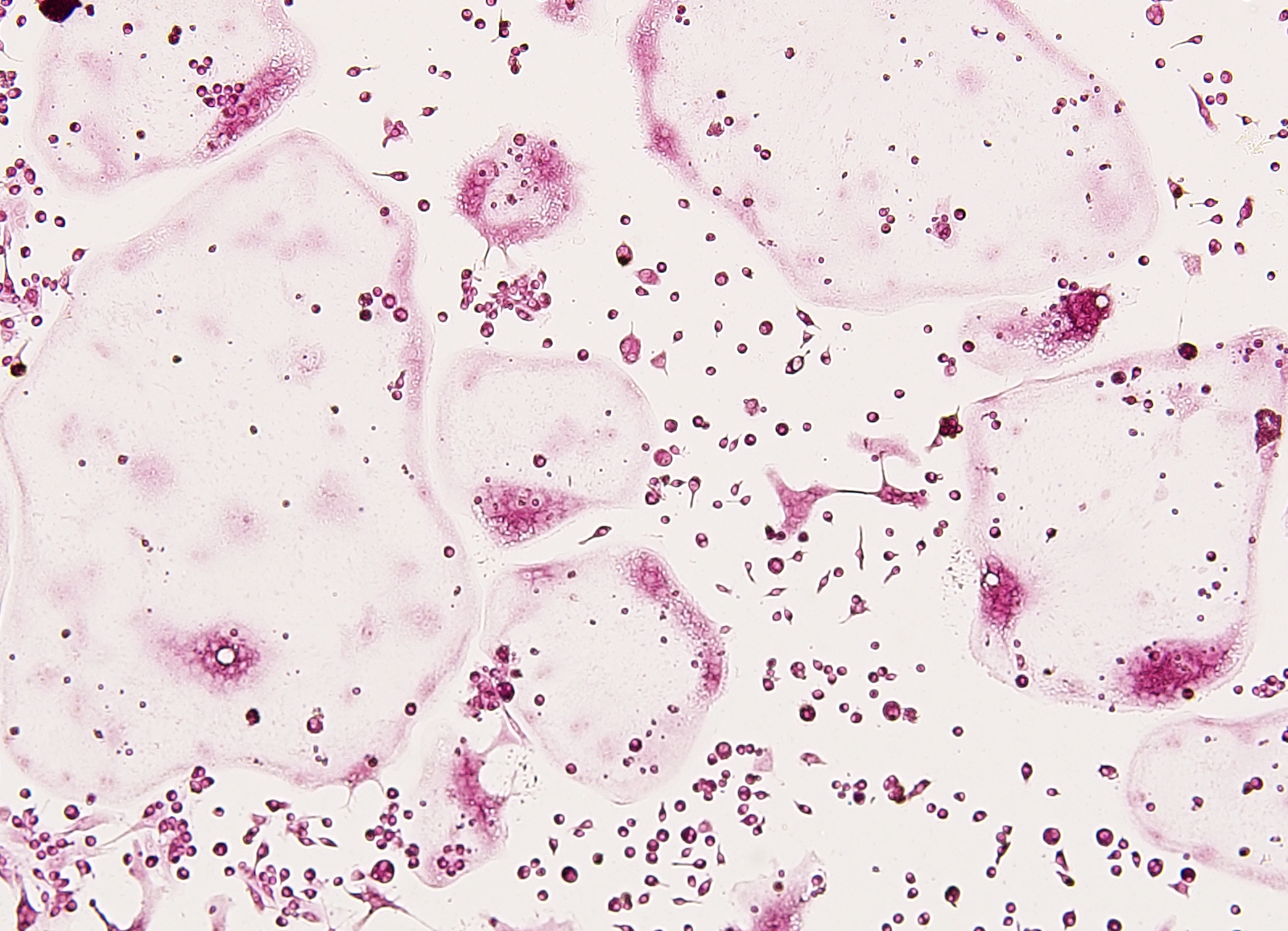

Supplement: Supplemental Information 2 [file peerj-11-14560-s002.zip › All raw data(including WB data) version 2/FIGURE 2/A/RANKL(+)-I(20).jpg]

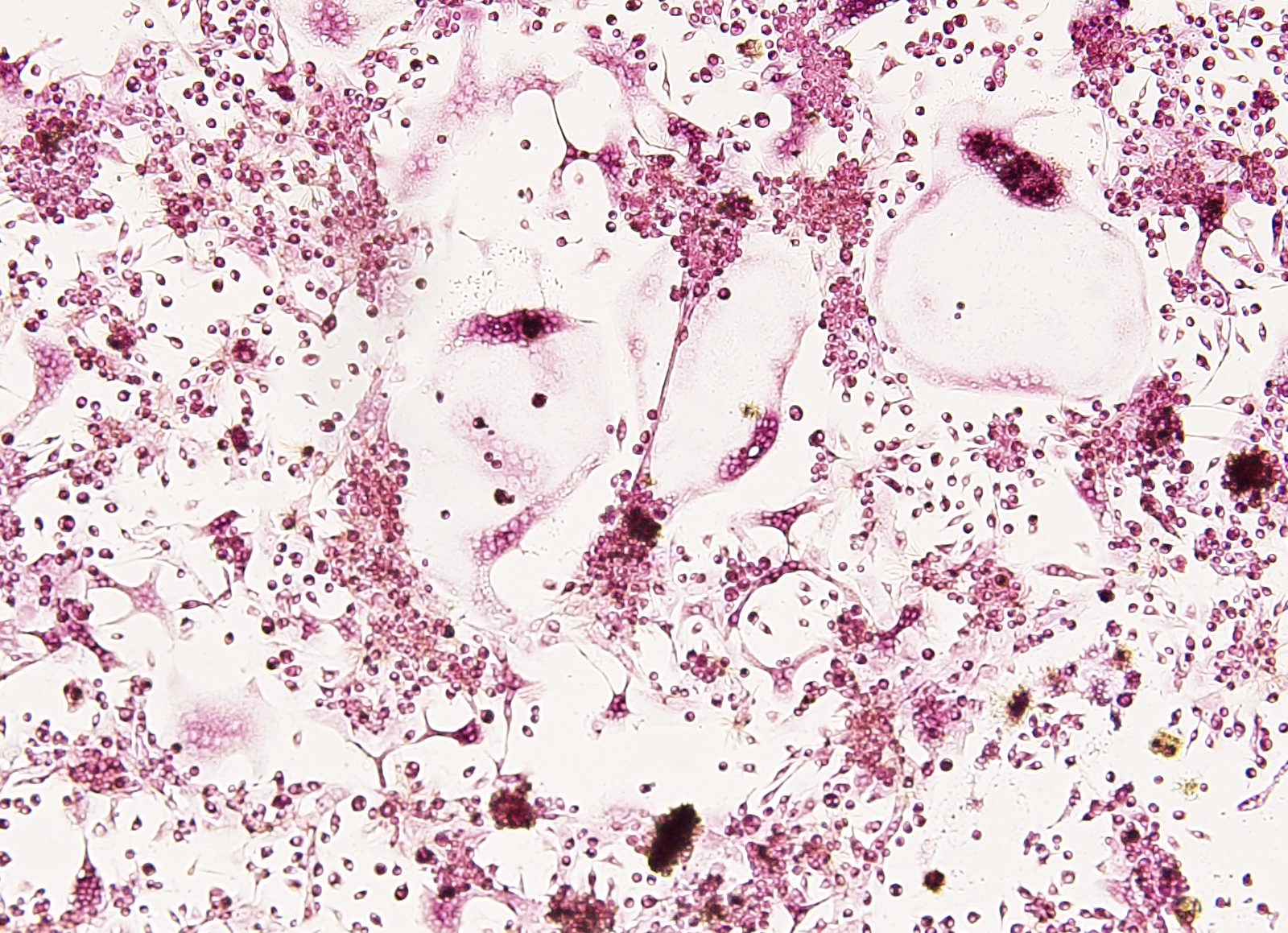

Supplement: Supplemental Information 2 [file peerj-11-14560-s002.zip › All raw data(including WB data) version 2/FIGURE 2/A/RANKL(+)-I(30).jpg]

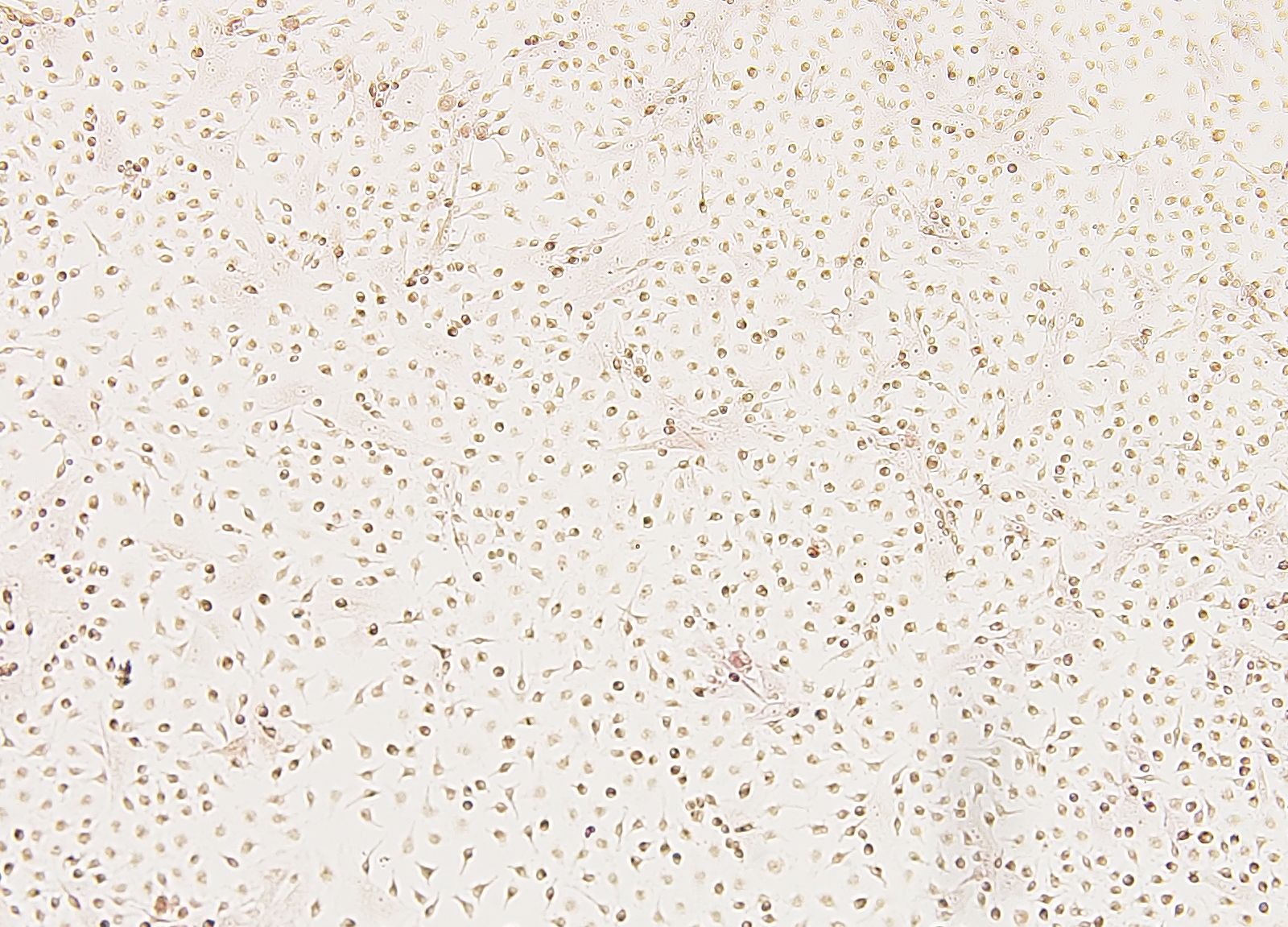

Supplement: Supplemental Information 2 [file peerj-11-14560-s002.zip › All raw data(including WB data) version 2/FIGURE 2/A/RANKL(-).jpg]

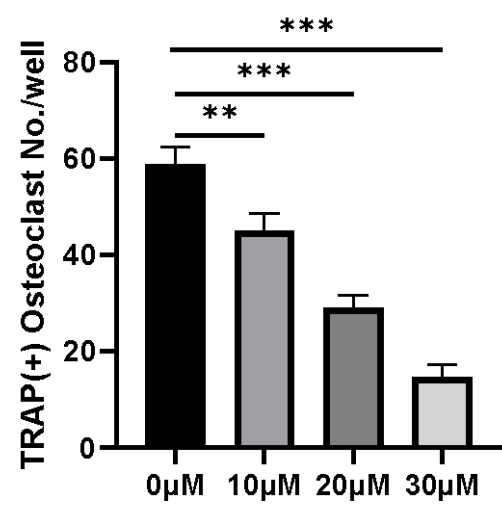

Supplement: Supplemental Information 2 [file peerj-11-14560-s002.zip › All raw data(including WB data) version 2/FIGURE 2/B/FIGURE 2 B.pdf]

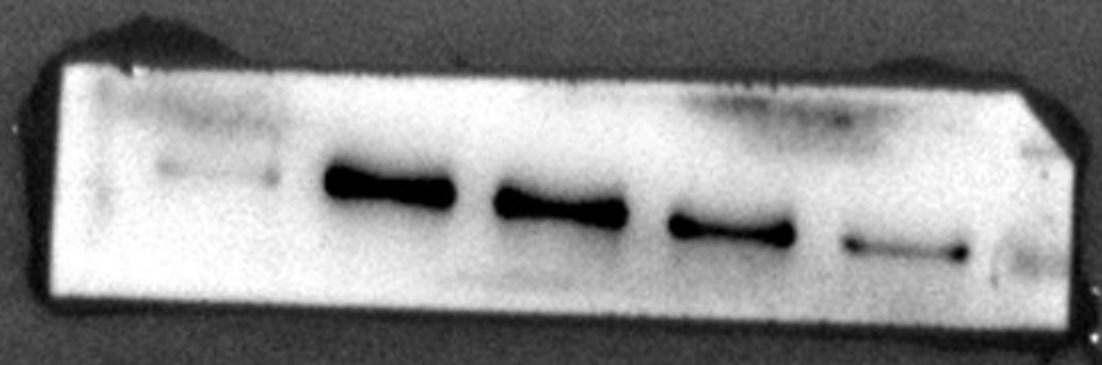

Supplement: Supplemental Information 2 [file peerj-11-14560-s002.zip › All raw data(including WB data) version 2/FIGURE 2/C(All raw WB data)/1 NFATC1-Merge.pdf]

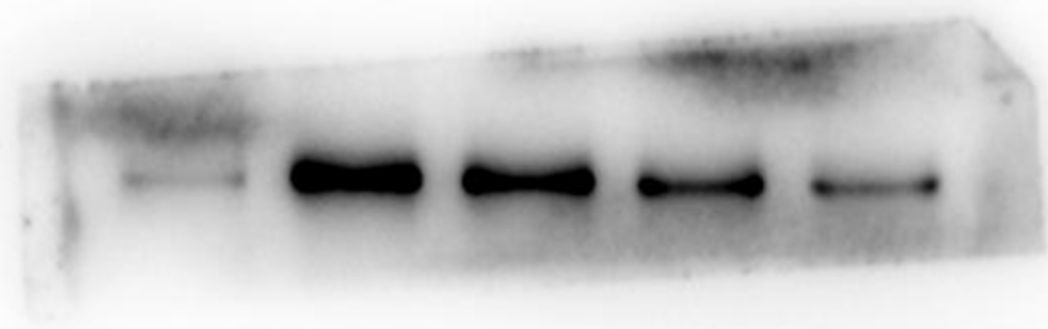

Supplement: Supplemental Information 2 [file peerj-11-14560-s002.zip › All raw data(including WB data) version 2/FIGURE 2/C(All raw WB data)/1 NFATC1.pdf]

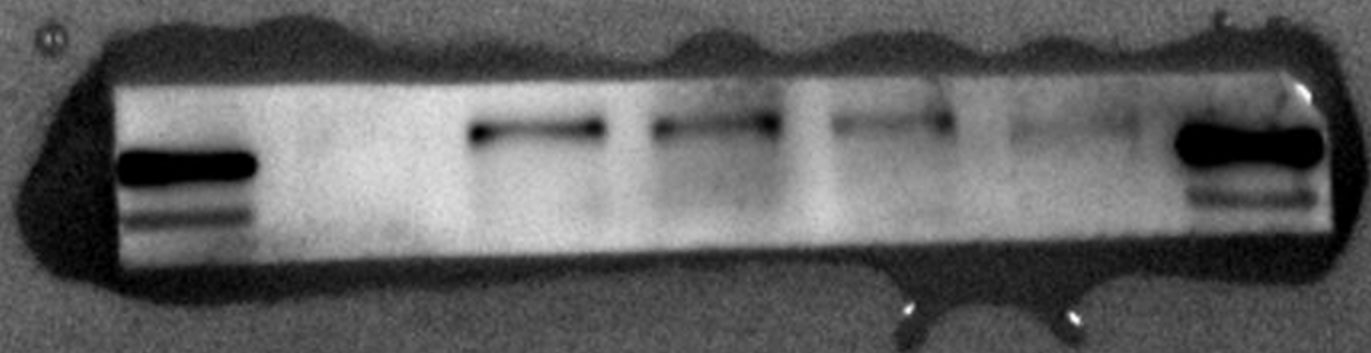

Supplement: Supplemental Information 2 [file peerj-11-14560-s002.zip › All raw data(including WB data) version 2/FIGURE 2/C(All raw WB data)/2 MMP9-Merge.pdf]

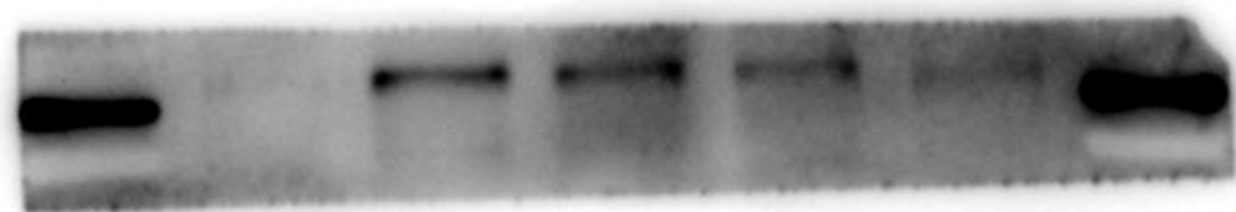

Supplement: Supplemental Information 2 [file peerj-11-14560-s002.zip › All raw data(including WB data) version 2/FIGURE 2/C(All raw WB data)/2 MMP9.pdf]

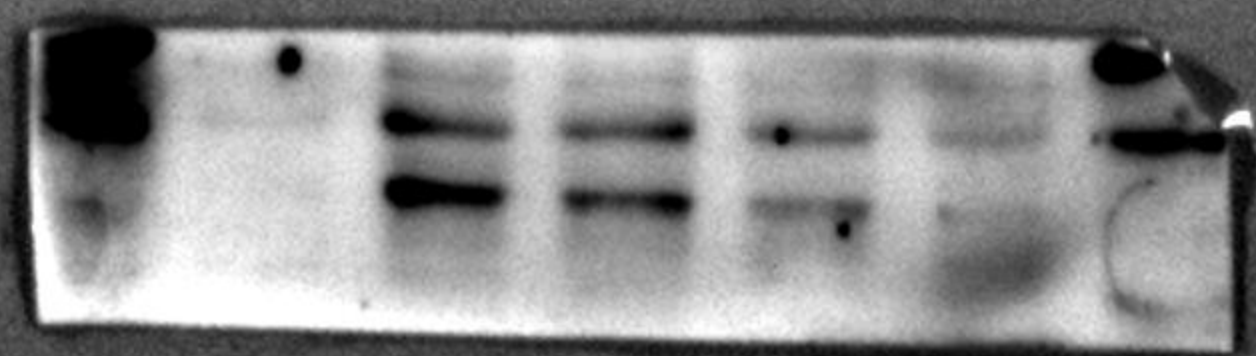

Supplement: Supplemental Information 2 [file peerj-11-14560-s002.zip › All raw data(including WB data) version 2/FIGURE 2/C(All raw WB data)/3 CTSK-Merge.pdf]

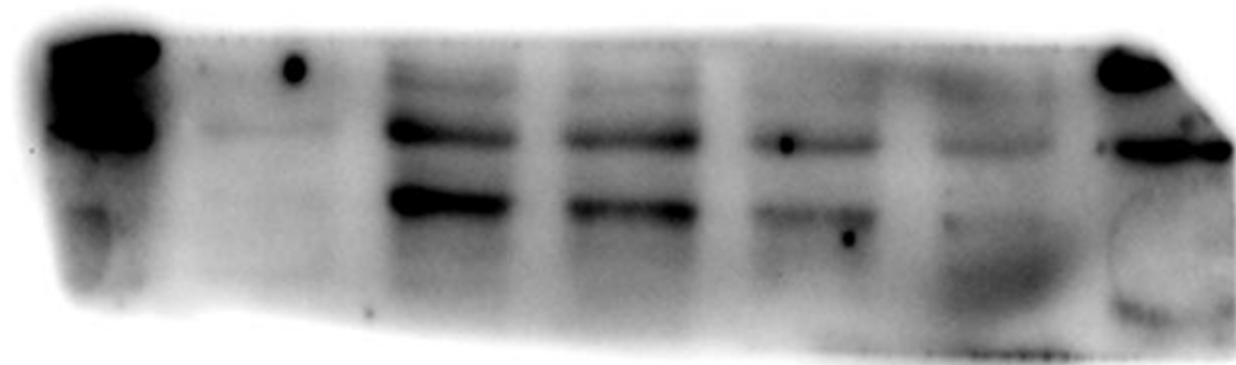

Supplement: Supplemental Information 2 [file peerj-11-14560-s002.zip › All raw data(including WB data) version 2/FIGURE 2/C(All raw WB data)/3 CTSK.pdf]

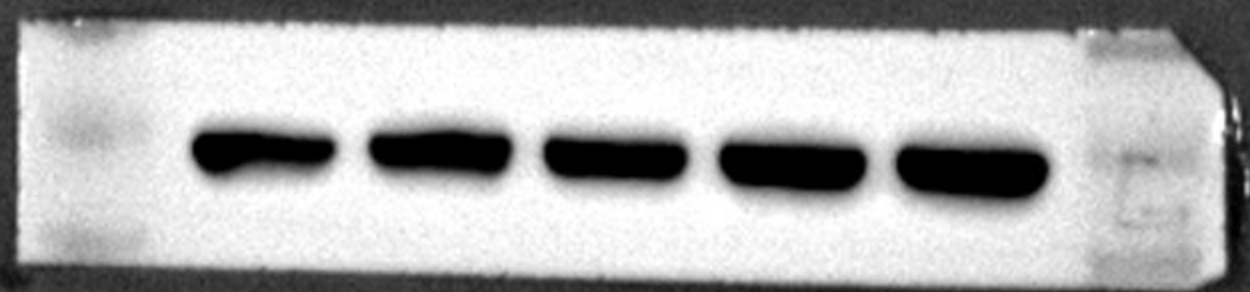

Supplement: Supplemental Information 2 [file peerj-11-14560-s002.zip › All raw data(including WB data) version 2/FIGURE 2/C(All raw WB data)/4 GAPDH-Merge.pdf]

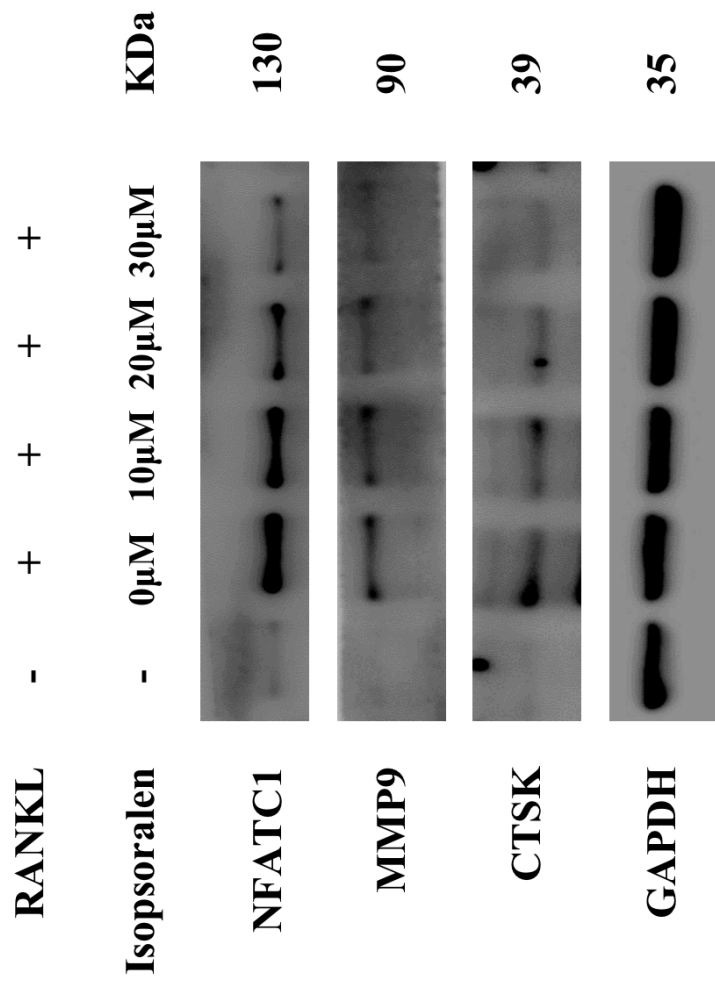

Supplement: Supplemental Information 2 [file peerj-11-14560-s002.zip › All raw data(including WB data) version 2/FIGURE 2/C(All raw WB data)/FIGURE 2 C.pdf]

130kDa

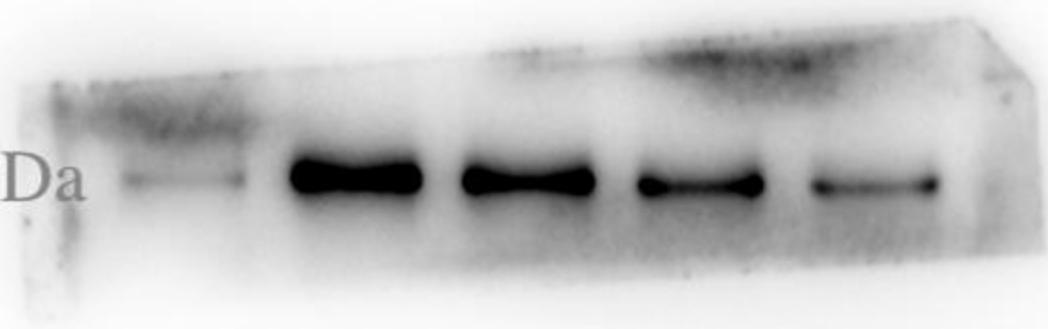

Supplement: Supplemental Information 2 [file peerj-11-14560-s002.zip › All raw data(including WB data) version 2/FIGURE 2/C(All raw WB data)/indicated marker version/1 NFATC1.pdf]

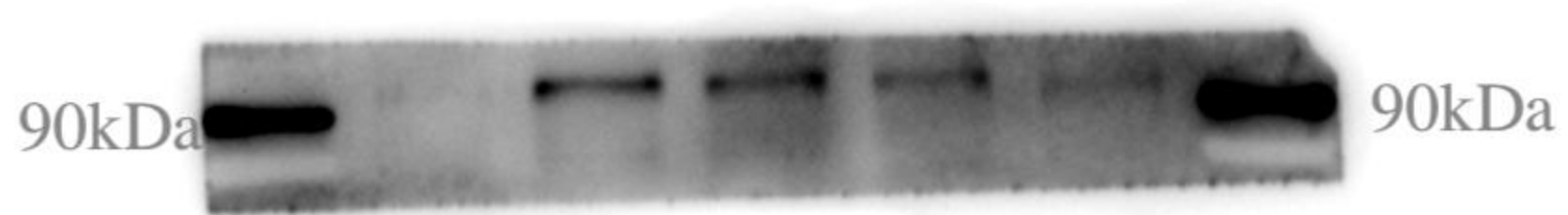

Supplement: Supplemental Information 2 [file peerj-11-14560-s002.zip › All raw data(including WB data) version 2/FIGURE 2/C(All raw WB data)/indicated marker version/2 MMP9.pdf]

39kDa

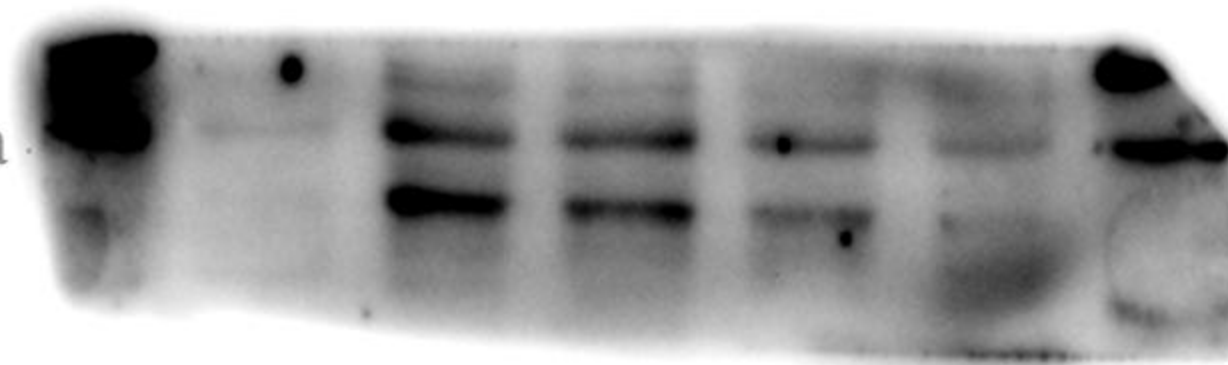

39kDa

Supplement: Supplemental Information 2 [file peerj-11-14560-s002.zip › All raw data(including WB data) version 2/FIGURE 2/C(All raw WB data)/indicated marker version/3 CTSK.pdf]

35kDa 35kDa

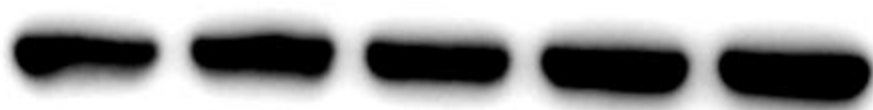

Supplement: Supplemental Information 2 [file peerj-11-14560-s002.zip › All raw data(including WB data) version 2/FIGURE 2/C(All raw WB data)/indicated marker version/4 GAPDH.pdf]

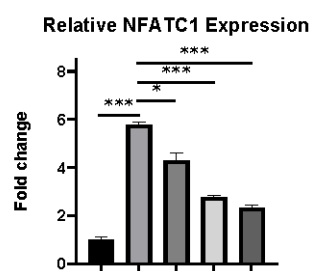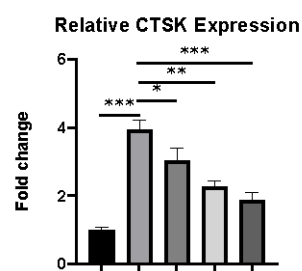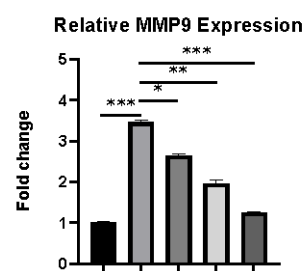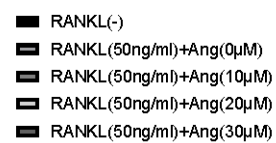

Supplement: Supplemental Information 2 [file peerj-11-14560-s002.zip › All raw data(including WB data) version 2/FIGURE 2/D/FIGURE 2 D.pdf]

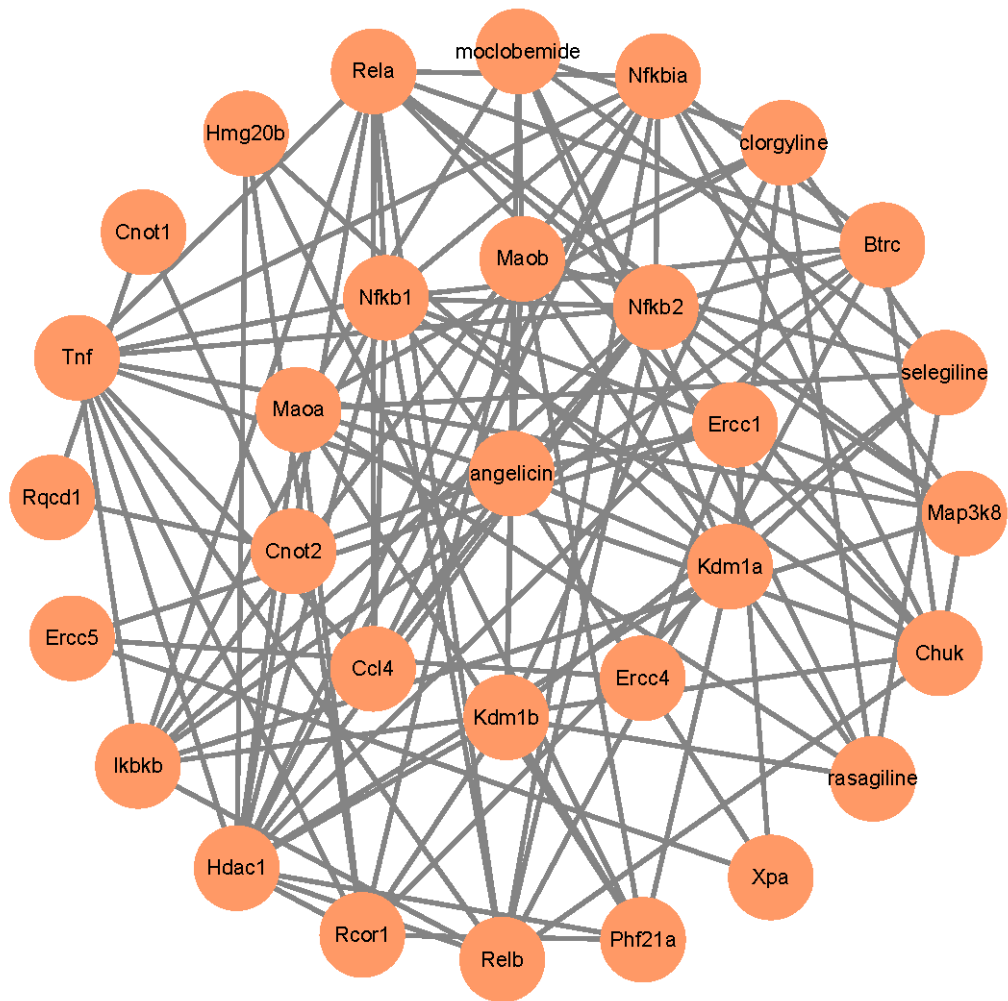

Supplement: Supplemental Information 2 [file peerj-11-14560-s002.zip › All raw data(including WB data) version 2/FIGURE 3/A/FIGURE 3 A.pdf]

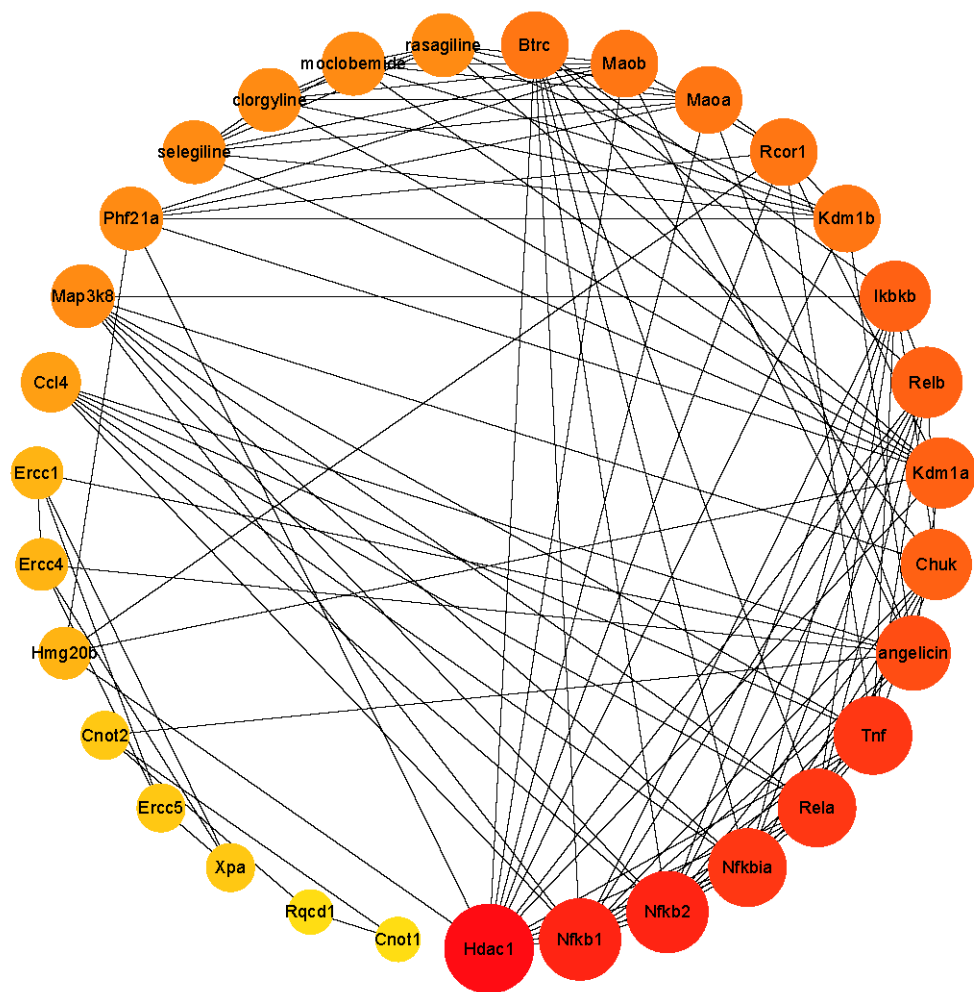

Supplement: Supplemental Information 2 [file peerj-11-14560-s002.zip › All raw data(including WB data) version 2/FIGURE 3/B/FIGURE 3 B.pdf]

# KEGG Pathway Enrichment

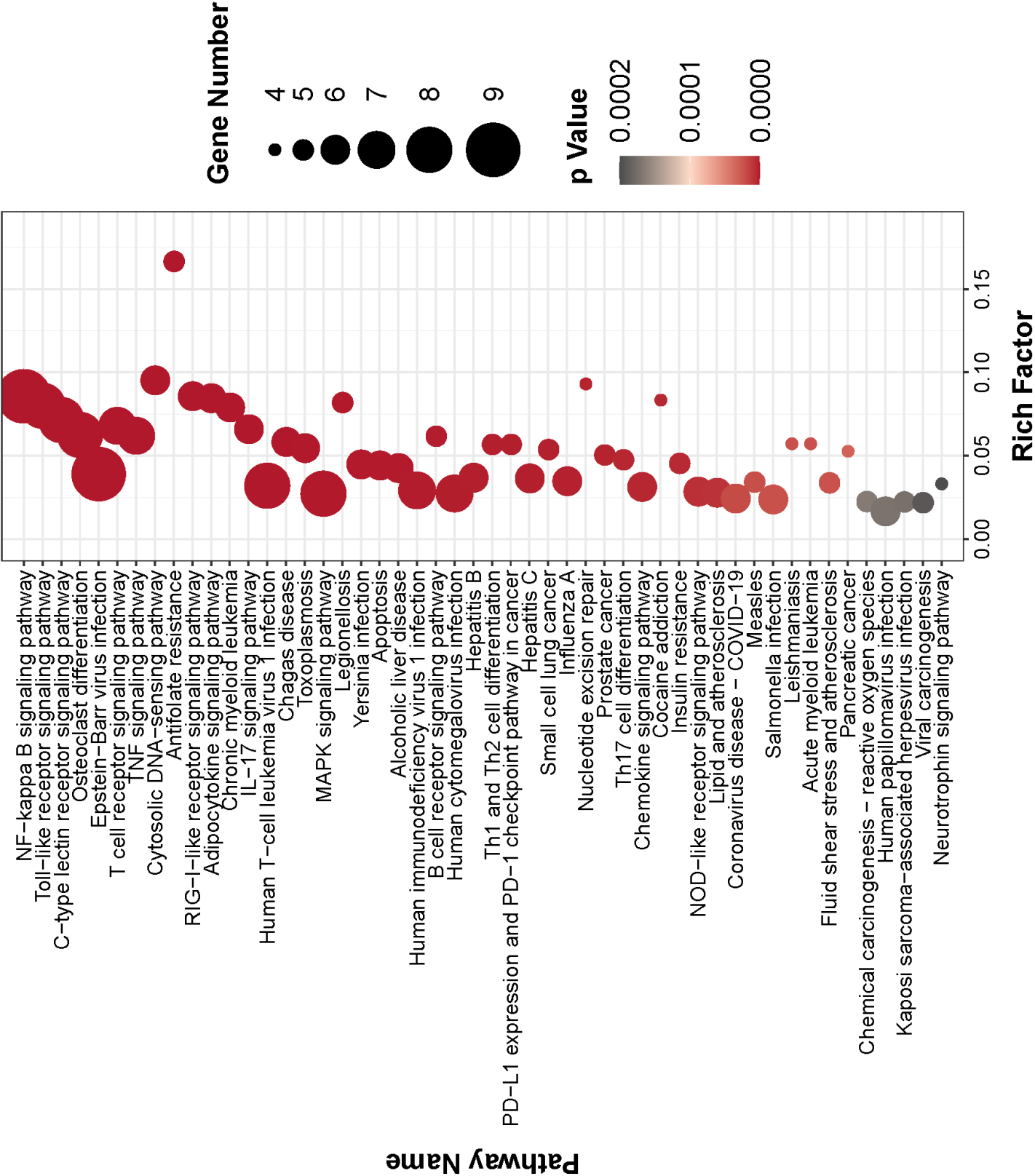

Supplement: Supplemental Information 2 [file peerj-11-14560-s002.zip › All raw data(including WB data) version 2/FIGURE 3/C/FIGURE 3 C.pdf]

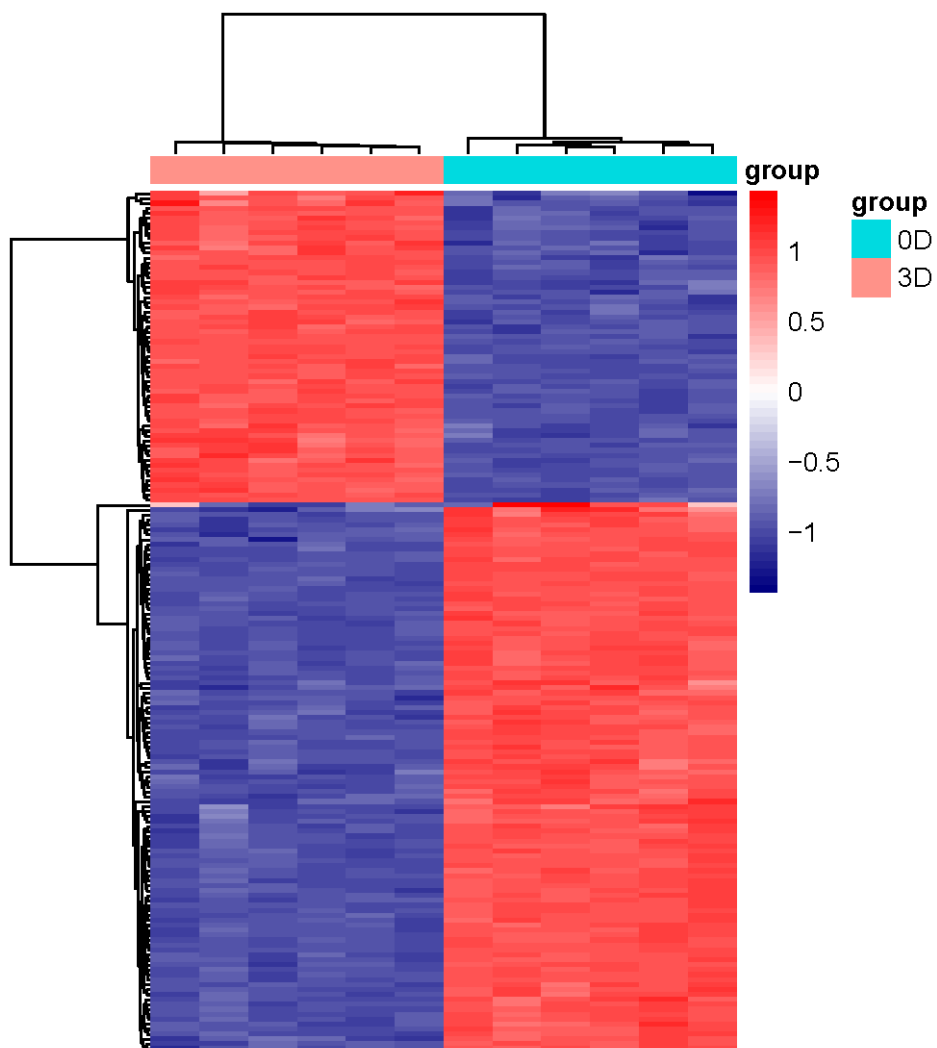

Supplement: Supplemental Information 2 [file peerj-11-14560-s002.zip › All raw data(including WB data) version 2/FIGURE 3/D/FIGURE 3 D.pdf]

# KEGG Pathway Enrichment

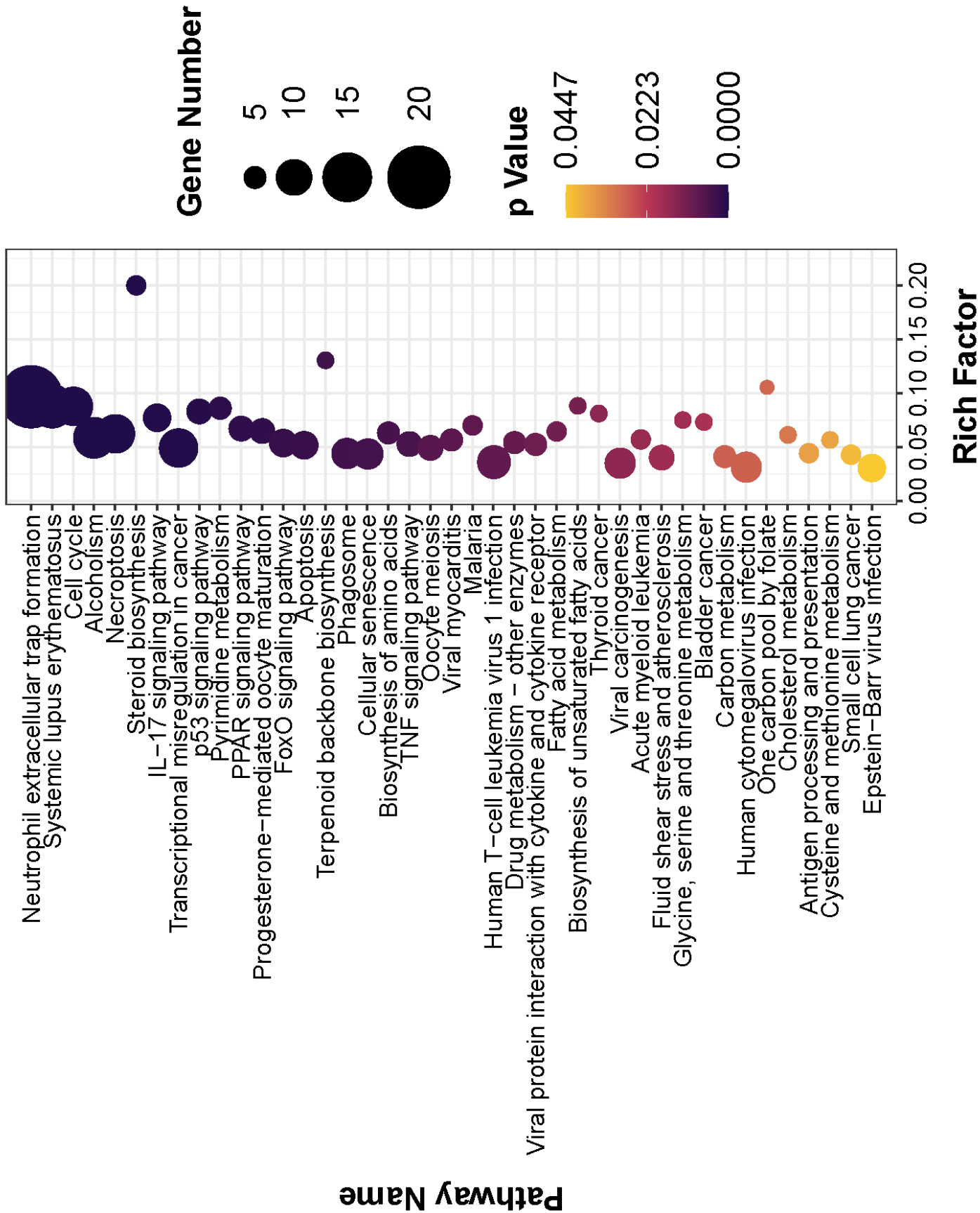

Supplement: Supplemental Information 2 [file peerj-11-14560-s002.zip › All raw data(including WB data) version 2/FIGURE 3/E/FIGURE 3 E.pdf]

KEGG Pathway

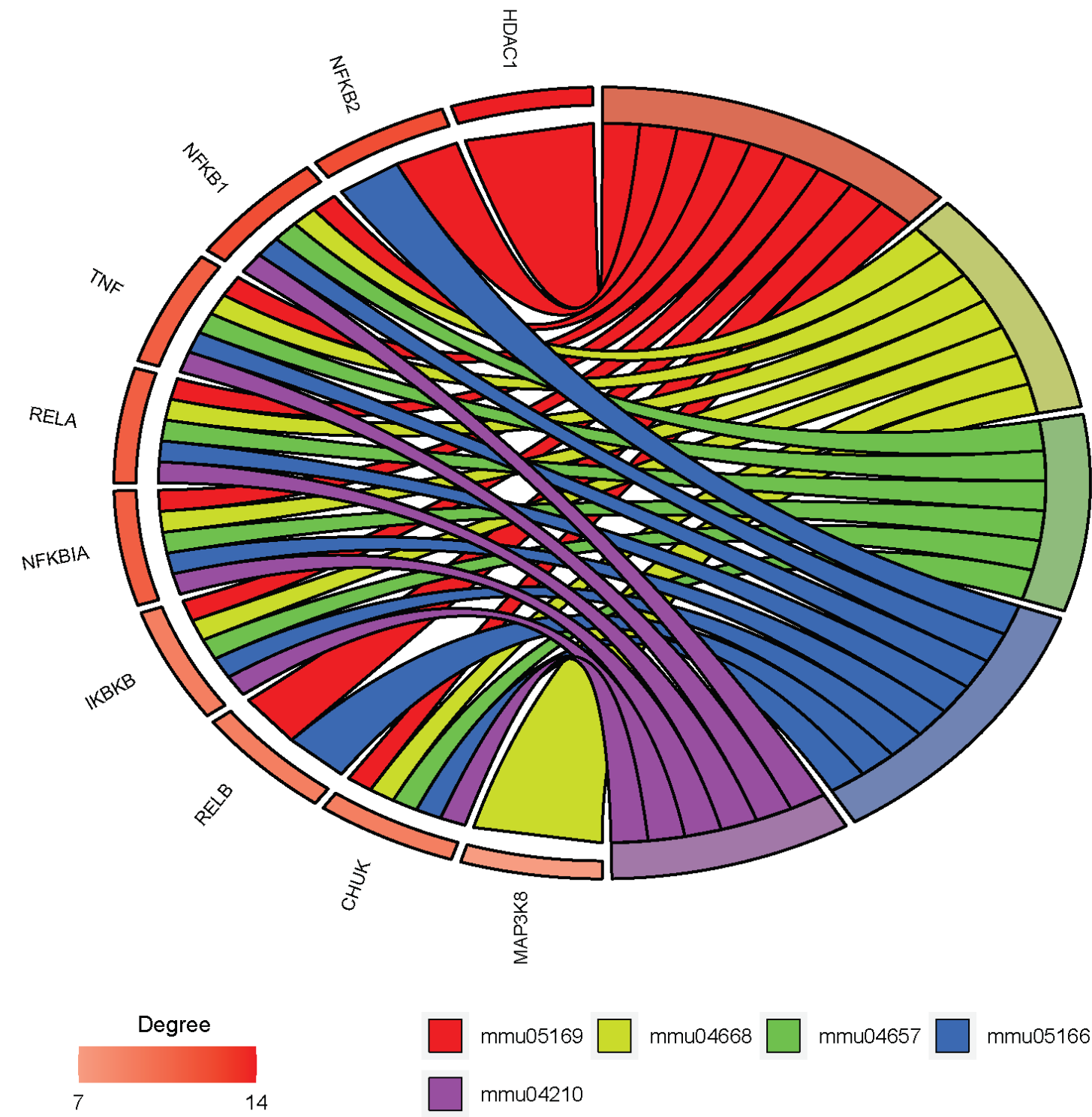

Supplement: Supplemental Information 2 [file peerj-11-14560-s002.zip › All raw data(including WB data) version 2/FIGURE 4/A/FIGURE 4 A.pdf]

# OSTEOCLAST DIFFERENTIATION

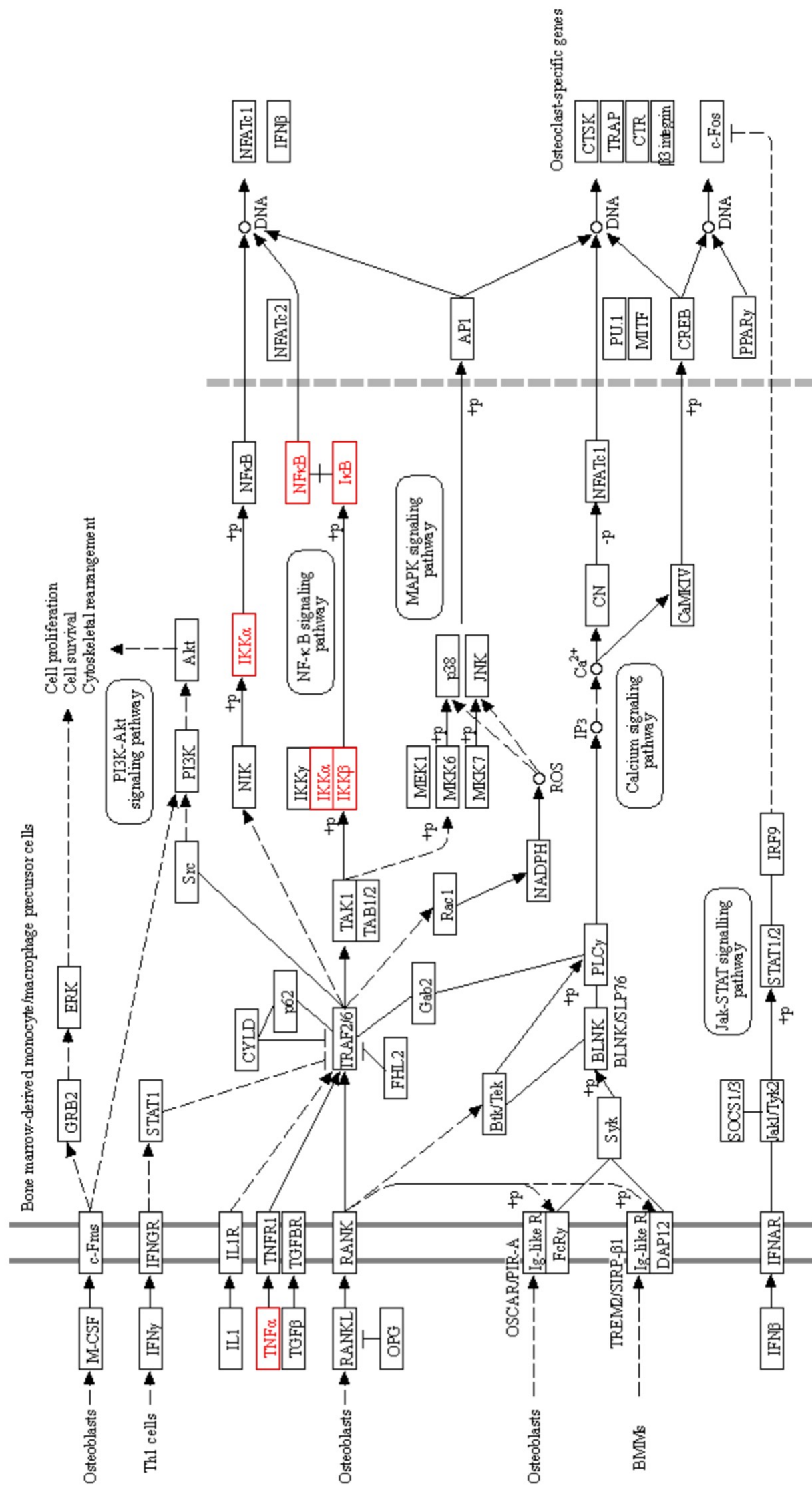

Supplement: Supplemental Information 2 [file peerj-11-14560-s002.zip › All raw data(including WB data) version 2/FIGURE 4/B/FIGURE 4 B.pdf]

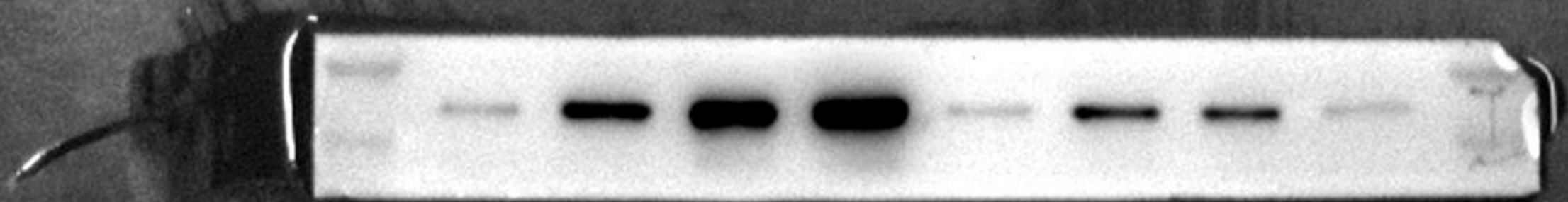

Supplement: Supplemental Information 2 [file peerj-11-14560-s002.zip › All raw data(including WB data) version 2/FIGURE 4/C(All raw WB data)/1 P-IKBA-Merge.pdf]

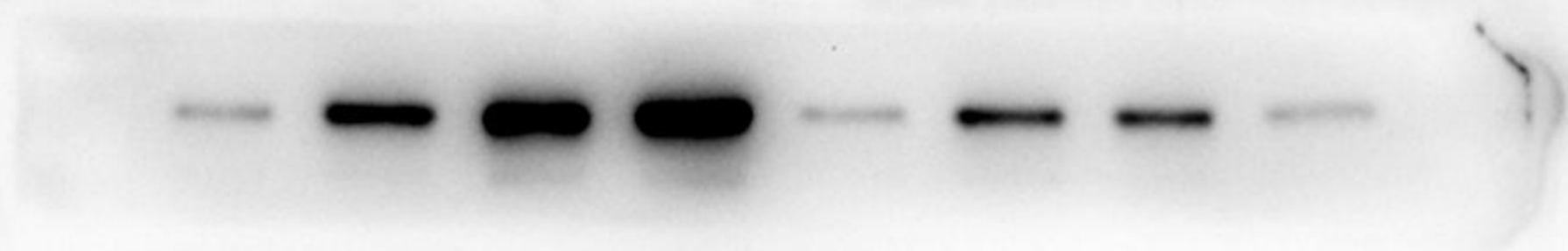

Supplement: Supplemental Information 2 [file peerj-11-14560-s002.zip › All raw data(including WB data) version 2/FIGURE 4/C(All raw WB data)/1 P-IKBA.pdf]

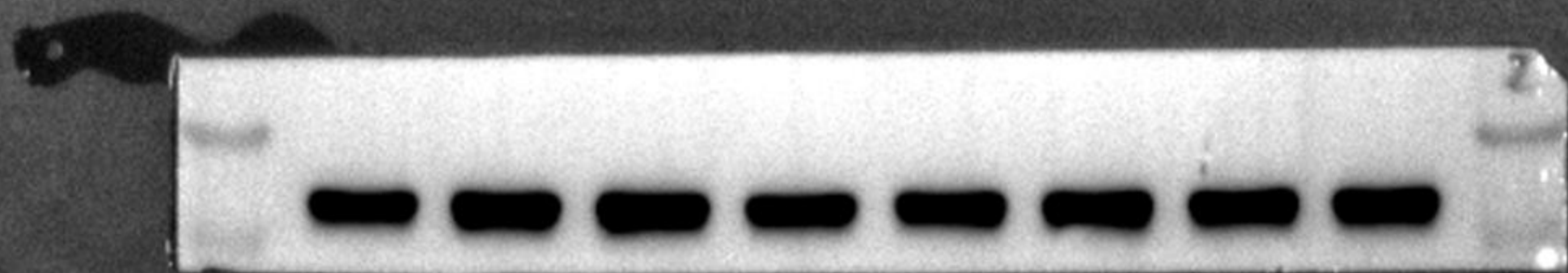

Supplement: Supplemental Information 2 [file peerj-11-14560-s002.zip › All raw data(including WB data) version 2/FIGURE 4/C(All raw WB data)/2 IKBA-Merge.pdf]

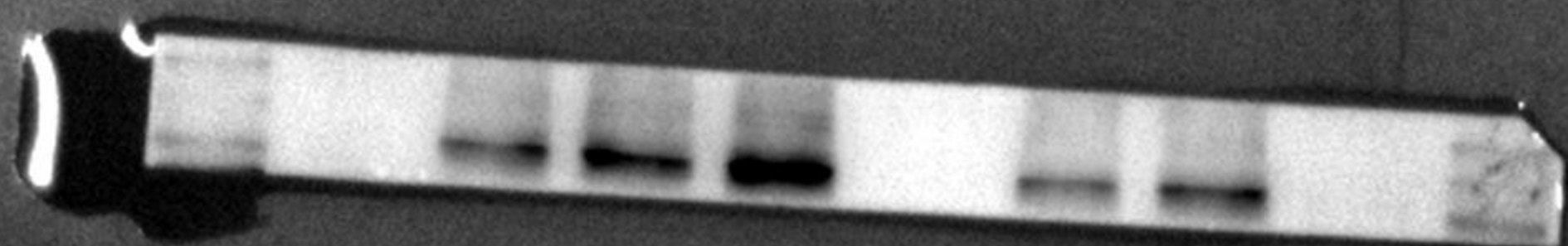

Supplement: Supplemental Information 2 [file peerj-11-14560-s002.zip › All raw data(including WB data) version 2/FIGURE 4/C(All raw WB data)/3 P-P50-Merge.pdf]

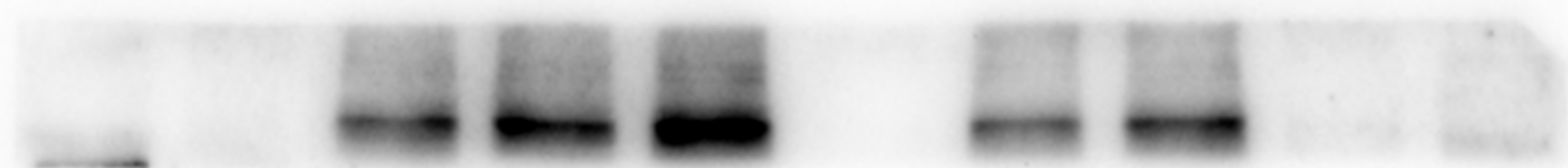

Supplement: Supplemental Information 2 [file peerj-11-14560-s002.zip › All raw data(including WB data) version 2/FIGURE 4/C(All raw WB data)/3 P-P50.pdf]

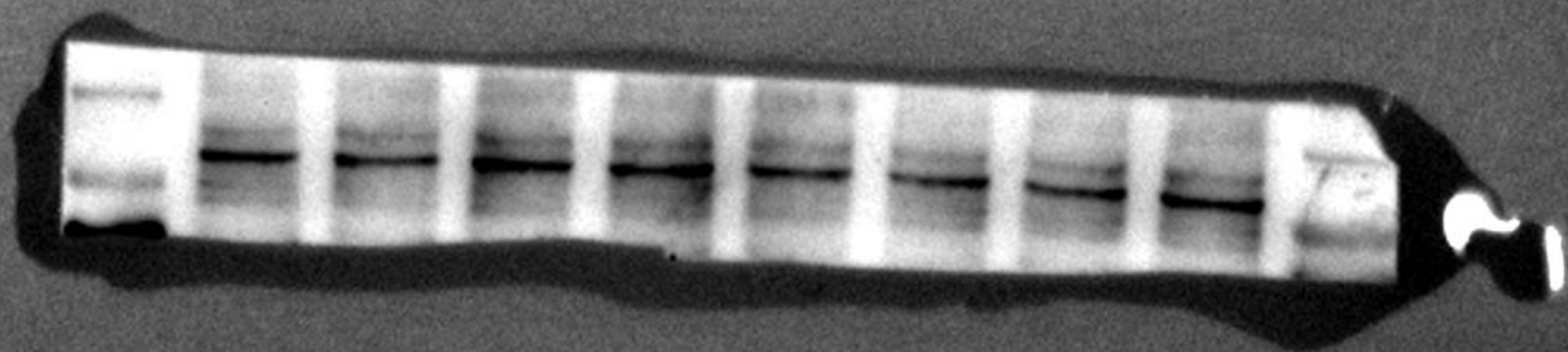

Supplement: Supplemental Information 2 [file peerj-11-14560-s002.zip › All raw data(including WB data) version 2/FIGURE 4/C(All raw WB data)/4 P50-Merge.pdf]

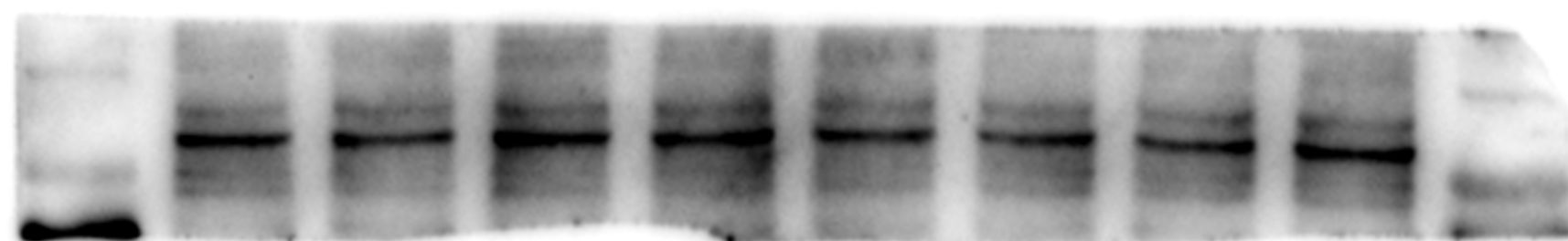

Supplement: Supplemental Information 2 [file peerj-11-14560-s002.zip › All raw data(including WB data) version 2/FIGURE 4/C(All raw WB data)/4 P50.pdf]

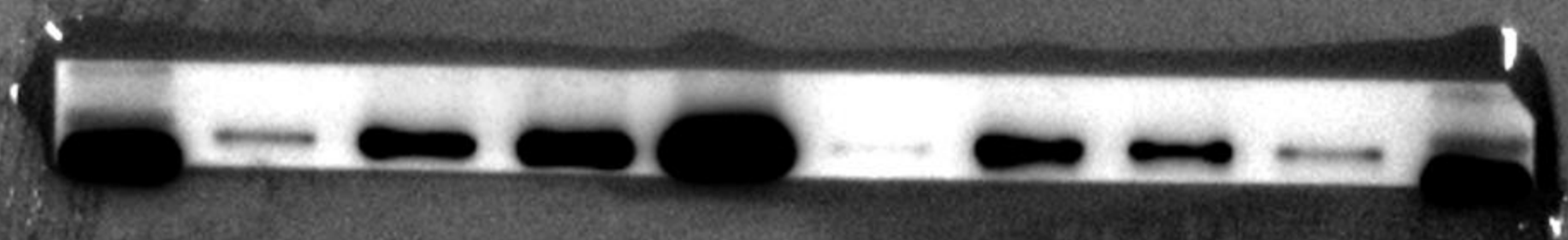

Supplement: Supplemental Information 2 [file peerj-11-14560-s002.zip › All raw data(including WB data) version 2/FIGURE 4/C(All raw WB data)/5 P-P65-Merge.pdf]

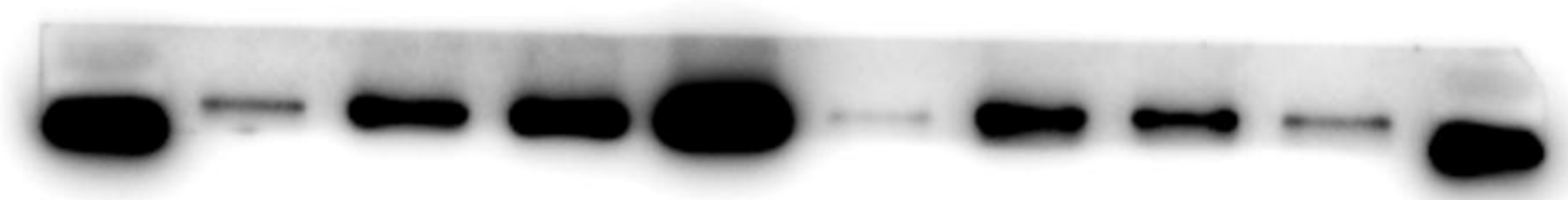

Supplement: Supplemental Information 2 [file peerj-11-14560-s002.zip › All raw data(including WB data) version 2/FIGURE 4/C(All raw WB data)/5 P-P65.pdf]

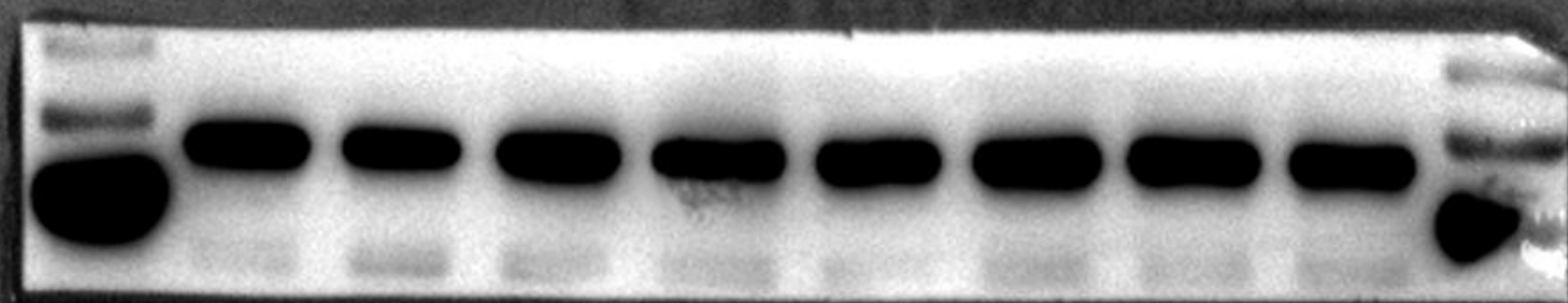

Supplement: Supplemental Information 2 [file peerj-11-14560-s002.zip › All raw data(including WB data) version 2/FIGURE 4/C(All raw WB data)/6 P65-Merge.pdf]

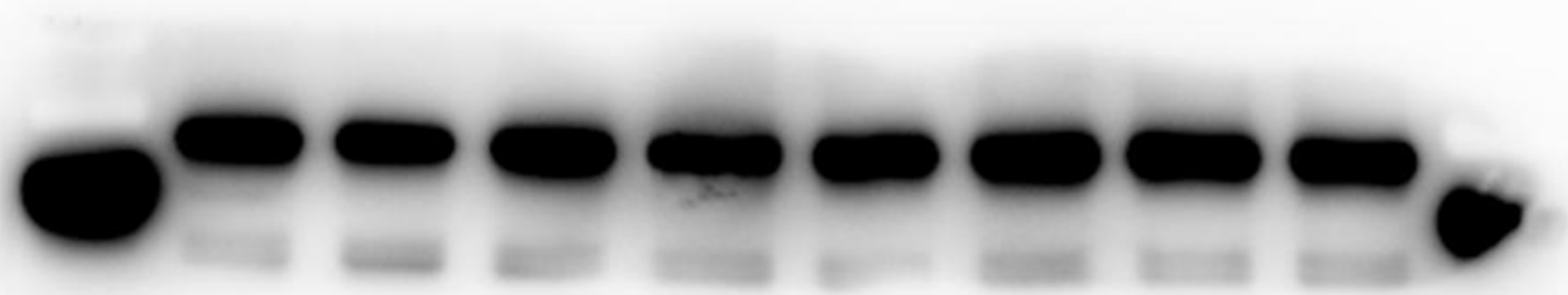

Supplement: Supplemental Information 2 [file peerj-11-14560-s002.zip › All raw data(including WB data) version 2/FIGURE 4/C(All raw WB data)/6 P65.pdf]

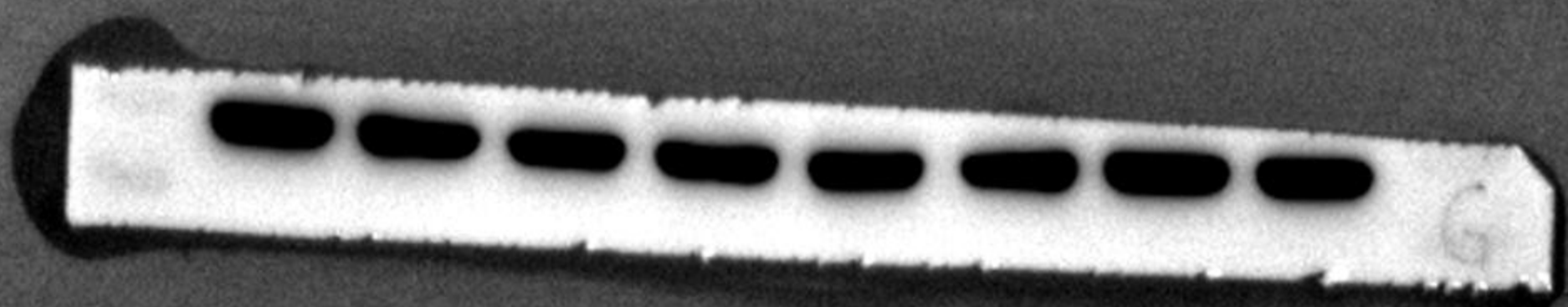

Supplement: Supplemental Information 2 [file peerj-11-14560-s002.zip › All raw data(including WB data) version 2/FIGURE 4/C(All raw WB data)/7 GAPDH-Merge.pdf]

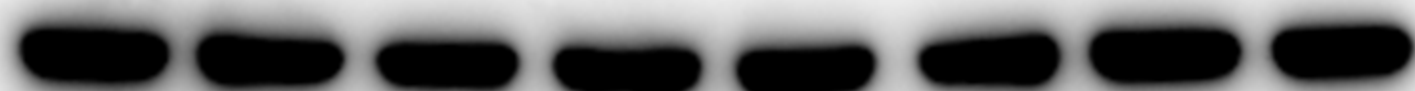

Supplement: Supplemental Information 2 [file peerj-11-14560-s002.zip › All raw data(including WB data) version 2/FIGURE 4/C(All raw WB data)/7 GAPDH.pdf]

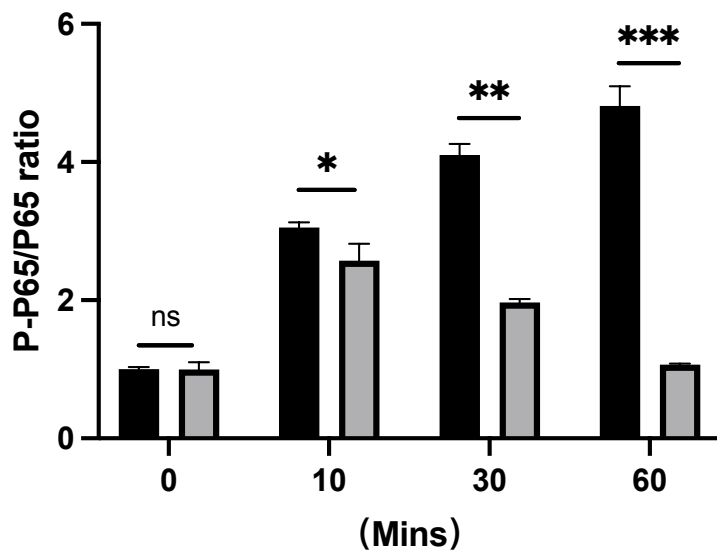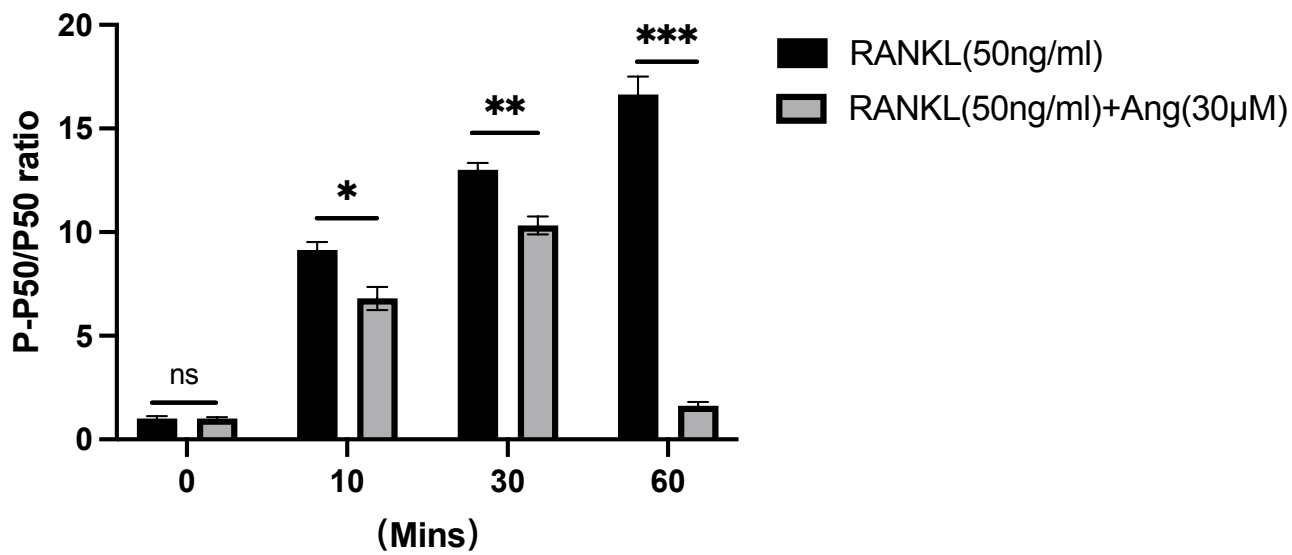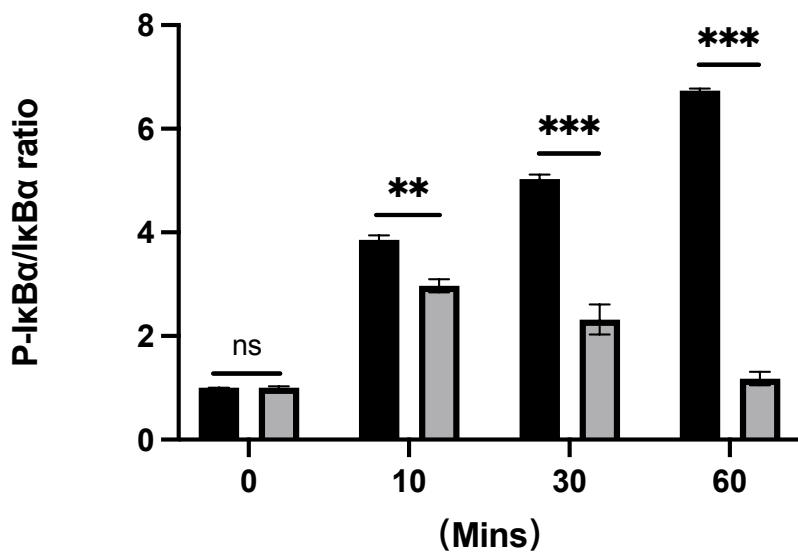

Supplement: Supplemental Information 2 [file peerj-11-14560-s002.zip › All raw data(including WB data) version 2/FIGURE 4/C(All raw WB data)/FIGURE 4 C.pdf]

40kDa

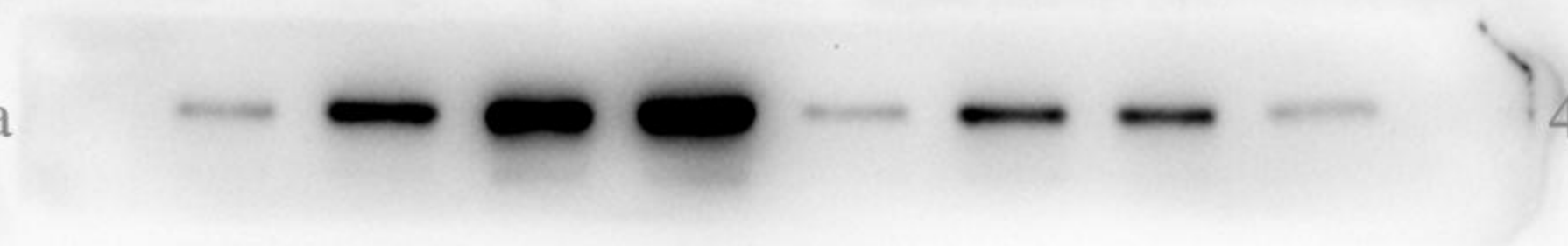

40kDa

Supplement: Supplemental Information 2 [file peerj-11-14560-s002.zip › All raw data(including WB data) version 2/FIGURE 4/C(All raw WB data)/indicated marker version/1 P-IKBA.pdf]

39kDa

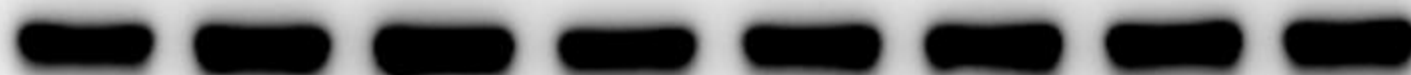

39kDa

Supplement: Supplemental Information 2 [file peerj-11-14560-s002.zip › All raw data(including WB data) version 2/FIGURE 4/C(All raw WB data)/indicated marker version/2 IKBA.pdf]

50kDa

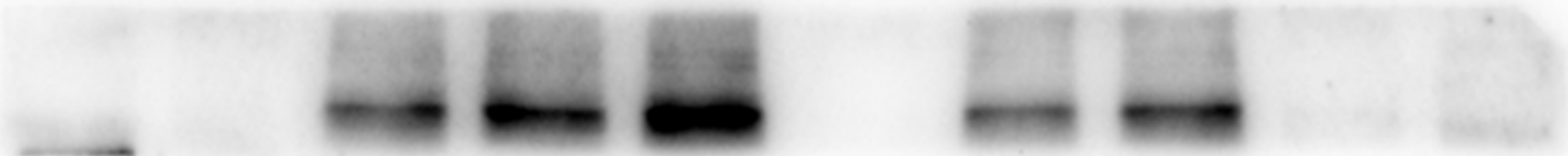

50kDa

Supplement: Supplemental Information 2 [file peerj-11-14560-s002.zip › All raw data(including WB data) version 2/FIGURE 4/C(All raw WB data)/indicated marker version/3 P-P50.pdf]

105kDa

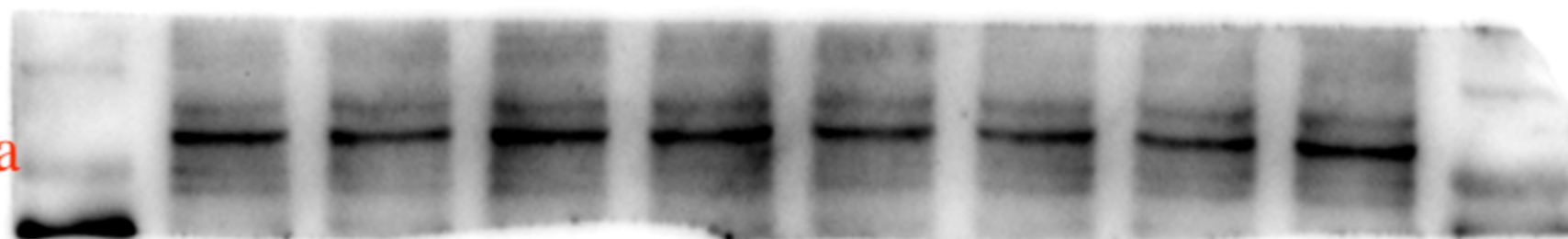

105kDa

Supplement: Supplemental Information 2 [file peerj-11-14560-s002.zip › All raw data(including WB data) version 2/FIGURE 4/C(All raw WB data)/indicated marker version/4 P50.pdf]

65kDa

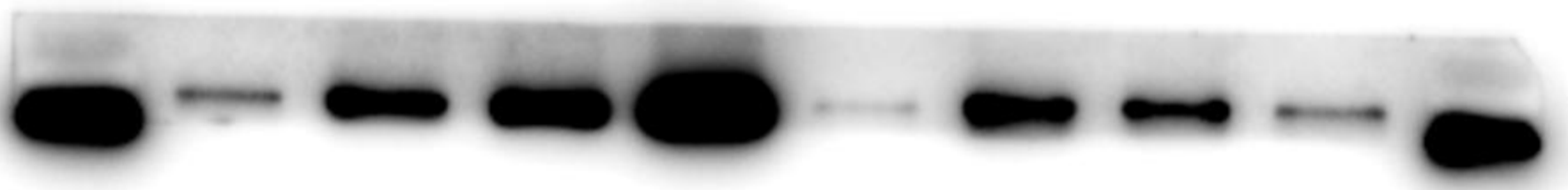

65kDa

Supplement: Supplemental Information 2 [file peerj-11-14560-s002.zip › All raw data(including WB data) version 2/FIGURE 4/C(All raw WB data)/indicated marker version/5 P-P65.pdf]

65kDa

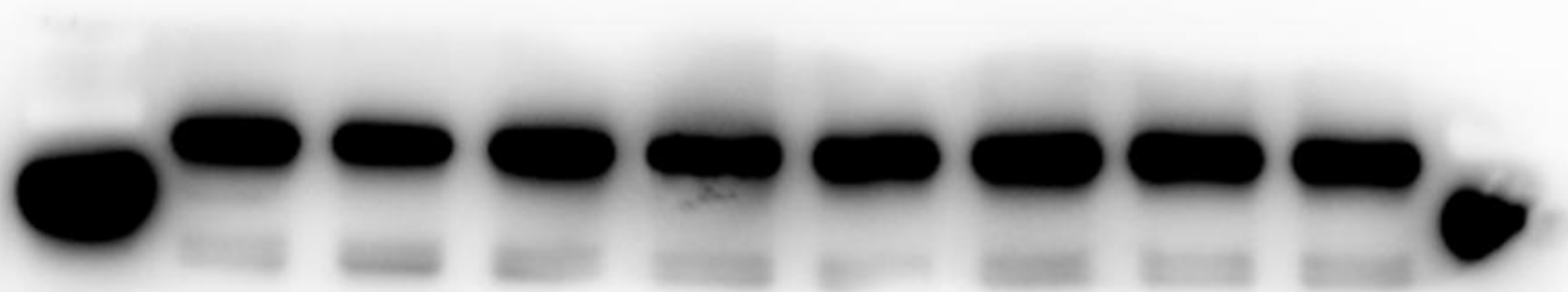

65kDa

Supplement: Supplemental Information 2 [file peerj-11-14560-s002.zip › All raw data(including WB data) version 2/FIGURE 4/C(All raw WB data)/indicated marker version/6 P65.pdf]

35kDa

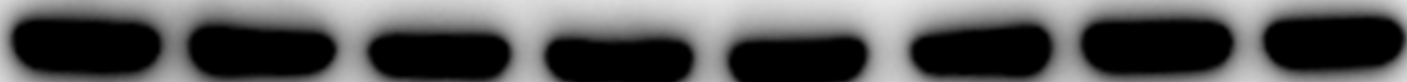

35kDa

Supplement: Supplemental Information 2 [file peerj-11-14560-s002.zip › All raw data(including WB data) version 2/FIGURE 4/C(All raw WB data)/indicated marker version/7 GAPDH.pdf]
